# Phenylalanine–tyrosine–catecholamine axis disorders: pathways, molecular diagnosis, therapeutics, and emerging translational monitoring technologies

**DOI:** 10.3389/fmolb.2026.1767655

**Published:** 2026-05-15

**Authors:** Martina Isabella Armas Samaniego, Benjamin Arias-Almeida, Andrés León-Piñeiros, Jorge G. Figueroa, Andrea Vargas-Freire, Melissa López, Natalí Solano-Cueva, Juan Carlos Collantes, María de Lourdes Huiracocha-Tutiven, Gabriele Davide Bigoni-Ordóñez, Juan Carlos Pozo-Palacios, Vanessa Romero

**Affiliations:** 1 Departamento de Biotecnología, Colegio de Ciencias Biológicas y Ambientales, Universidad San Francisco de Quito, Quito, Pichincha, Ecuador; 2 Escuela de Medicina, Colegio de Ciencias de la Salud, Universidad San Francisco de Quito, Quito, Pichincha, Ecuador; 3 Instituto de Microbiología, Colegio de Ciencias Biológicas y Ambientales, Universidad San Francisco de Quito, Quito, Pichincha, Ecuador; 4 Departamento de Química, Facultad de Ciencias Exactas y Naturales, Universidad Técnica Particular de Loja, Loja, Ecuador; 5 Carrera de Medicina, Facultad de Ciencias Médicas, Universidad de Cuenca, Cuenca, Ecuador; 6 Carrera de Laboratorio Clínico, Facultad de Ciencias Médicas, Universidad de Cuenca, Cuenca, Ecuador

**Keywords:** aromatic l-amino acid decarboxylase deficiency, biochemical diagnosis, catecholamine biosynthesis, dopamine beta-hydroxylase deficiency, molecular diagnosis, phenylalanine hydroxylase deficiency, tyrosine hydroxylase deficiency

## Abstract

Disorders of the phenylalanine–tyrosine–catecholamine axis are a clinically relevant group of neurometabolic conditions in which pathogenic variants in key enzymes impair dopamine and norepinephrine biosynthesis. Patients may present with movement disorders, autonomic dysfunction, developmental delay, and related neurobehavioral manifestations. In this narrative review, we synthesize the main enzymatic defects across the axis, focusing on phenylalanine hydroxylase, tyrosine hydroxylase, aromatic L-amino acid decarboxylase, and dopamine beta-hydroxylase. We describe how diagnostic practice has evolved from isolated biochemical assays to integrated approaches that link clinical phenotyping with targeted biochemical profiling and molecular confirmation. Genetic testing now supports diagnosis, treatment planning, and family counseling, while chromatographic and mass spectrometry-based methods remain essential for quantifying amino acids and neurotransmitter-related metabolites. We also discuss emerging biosensor-based strategies as a potential route to decentralized and minimally invasive monitoring.

## 1 Introduction

Inborn errors affecting the phenylalanine–tyrosine–catecholamine axis represent a clinically important subset of inherited metabolic diseases. Pathogenic variants in this pathway impair dopamine and norepinephrine production by altering precursor availability or key enzymatic steps in catecholamine biosynthesis, leading to neurometabolic phenotypes characterized by movement abnormalities, autonomic dysfunction, and neurodevelopmental impairment (Opladen et al., 2016). These disorders show marked clinical heterogeneity. Tyrosine hydroxylase deficiency (TH), for example, may present as dopamine-responsive dystonia, early-onset parkinsonism, or a more complex movement disorder (Stepien et al., 2021). Other defects in this pathway may also lead to developmental delay, autonomic manifestations, and broader neurobehavioral abnormalities, reflecting the diverse consequences of impaired catecholamine synthesis (Brennenstuhl et al., 2019).

**FIGURE 1:**
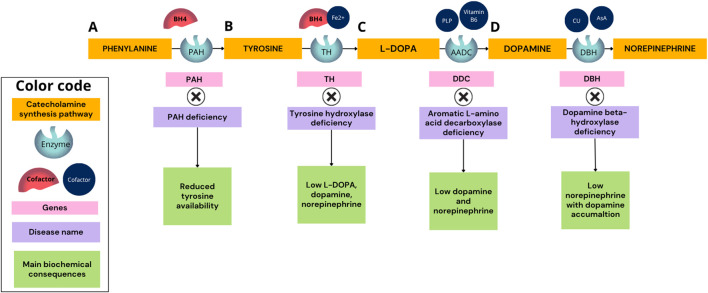
Catecholamine biosynthesis pathway and associated enzymatic deficiencies. The catecholamine biosynthesis pathway proceeds from phenylalanine to norepinephrine through sequential enzymatic steps catalyzed by phenylalanine hydroxylase (PAH), tyrosine hydroxylase (TH), aromatic L-amino acid decarboxylase (AADC/DDC), and dopamine beta-hydroxylase (DBH). **(A)** PAH deficiency results in phenylketonuria (PKU), characterized by reduced conversion of phenylalanine to tyrosine and consequent decreased tyrosine availability. **(B)** Tyrosine hydroxylase deficiency leads to impaired L-DOPA production, resulting in reduced dopamine and norepinephrine levels. **(C)** AADC deficiency disrupts the conversion of L-DOPA to dopamine, causing combined dopamine and norepinephrine deficiency and a spectrum of severe neurological manifestations. **(D)** DBH deficiency impairs the conversion of dopamine to norepinephrine, leading to low norepinephrine levels with dopamine accumulation, typically associated with autonomic dysfunction such as severe orthostatic hypotension. Cofactors required for each enzymatic step are indicated: tetrahydrobiopterin (BH4) for PAH and TH, pyridoxal phosphate (PLP, vitamin B6) for AADC, and ascorbate (vitamin C) and copper (Cu) for DBH.

Despite advances in diagnostic approaches, these conditions remain underdiagnosed because of overlapping neurological features, phenotypic variability, and limited access to specialized biochemical and molecular testing. These barriers are particularly relevant in low- and middle-income settings, where centralized genetic and metabolic services may delay diagnosis and restrict access to targeted treatment and specialized care (Aguirre et al., 2024a; Baxter et al., 2023; Georgiou et al., 2024; Kaufmann et al., 2018). This review focuses on the core enzymatic defects within this axis, with particular emphasis on Phenylalanine hydroxylase (PAH) deficiency, TH deficiency, aromatic L-amino acid decarboxylase (AADC) deficiency, and dopamine beta-hydroxylase (DBH) deficiency, addressing their molecular basis, biochemical signatures, clinical manifestations, diagnostic strategies, and therapeutic implications (Figure 1).

## 2 Clinical and molecular foundations

This pathway-based framework links specific enzymatic defects to characteristic biochemical profiles and clinical manifestations, which helps guide diagnostic and treatment strategies (Table 1) (Brennenstuhl et al., 2019; Garg, 2016). PAH converts phenylalanine to tyrosine, thereby providing the precursor required for downstream catecholamine biosynthesis (Gnegy, 2012). TH catalyzes the rate-limiting step by converting tyrosine to L-3,4-dihidroxifenilalanina (L-DOPA), and AADC converts L-DOPA to dopamine GCH1, PTS, and SPR affect BH4 synthesis, whereas QDPR and PCBD1 affect BH4 recycling. Because BH4 serves as an essential cofactor for tyrosine hydroxylase, defects in these genes can impair dopamine biosynthesis upstream of AADC (Daubner et al., 2011; Himmelreich et al., 2019; Végh et al., 2016). DBH subsequently converts dopamine to norepinephrine, mainly in noradrenergic neurons and adrenal chromaffin cells (Robertson et al., 1991; Rush and Geffen, 1980). Because each enzymatic block affects precursor availability and downstream catecholamine production, it can generate a recognizable biochemical pattern that supports pathway-based differential. Defects in PAH, TH, AADC, or DBH therefore produce distinct neurometabolic phenotypes, including movement abnormalities, autonomic dysfunction, developmental delay, and related neurological manifestations diagnosis.

**TABLE 1:** Overall review of current treatments and patient management for phenylalanine-tyrosine-catecholamine axis disorders.

Disease	Current treatments	Patient management	Reference(s)
PAH deficiency	Low-phenylalanine diet, tyrosine supplementation, sapropterin (Kuvan), pegvaliase (Palynziq)	Regular monitoring of phenylalanine levels, nutritional support	Blau (2013), Van Wegberg et al. (2025), Blau et al. (2010)
TH Deficiency	Levodopa/carbidopa, dopamine agonists, MAO-B inhibitors	Neurological evaluation, physical and occupational therapy	Daubner et al. (2011), Bräutigam et al. (1999)
AADC Deficiency	Gene therapy, dopamine agonists, MAO-B inhibitors, vitamin B6, folinic acid	Multidisciplinary management, physical and occupational therapy	Wassenberg et al. (2017), Car ly Kempler Pflaum (2024), Gucuyener et al. (2014)
DBH Deficiency	Droxidopa, fludrocortisone, indomethacin, MAO-B inhibitors	Cardiovascular monitoring, management of autonomic symptoms	Wassenberg et al. (2021), Biaggioni and Robertson (1987), Ma et al. (1987)

## 3 Genetics of phenylalanine–tyrosine–catecholamine axis

This section focuses on PAH, TH, AADC, and DBH, emphasizing the affected gene, the type of functional impairment, the resulting biochemical signature, and the main clinical correlation (Table 2) (Brennenstuhl et al., 2019; KuseyriHübschmann et al., 2021).

**TABLE 2:** Key genes, variants, and clinical implications in the phenylalanine-tyrosine-catecholamine axis disorders.

Enzyme/Function	Gene	Pathway role	Key variants	Pathophysiology	Clinical phenotype/Impact	References
Phenylalanine hydroxylase	*PAH*	Phenylalanine → Tyrosine	Loss-of-function variants	Impaired conversion → phenylalanine accumulation	Phenylketonuria (PKU): intellectual disability, seizures if untreated	Hillert et al. (2020), Blau et al. (2010)
Tyrosine hydroxylase	*TH*	Tyrosine → L-DOPA (rate-limiting step)	Rare pathogenic variants	Reduced dopamine, norepinephrine synthesis	Dopa-responsive dystonia, hypotonia, developmental delay	Daubner et al. (2011), Stamelou et al. (2012)
Aromatic L-amino acid decarboxylase	*DDC*	L-DOPA → Dopamine	Pathogenic variants	Reduced dopamine and serotonin; precursor accumulation	Hypotonia, oculogyric crises, movement disorders, autonomic dysfunction	Gucuyener et al. (2014), Rizzi et al. (2022)
Dopamine β-hydroxylase	*DBH*	Dopamine → Norepinephrine	Biallelic loss-of-function variants	Absence of norepinephrine; dopamine accumulation	Severe orthostatic hypotension, ptosis, autonomic dysfunction	Robertson et al. (1991), Rush and Geffen (1980)
BH4 synthesis/recycling	*GCH1, PTS, SPR, QDPR, PCBD1*	Cofactor for TH and AADC	Pathogenic variants	Reduced cofactor availability → impaired monoamine synthesis	Dopa-responsive dystonia, neurotransmitter deficiency syndromes	Himmelreich et al. (2021), Kim et al. (2023), Orphanet (2026)

These monogenic disorders represent key nodes in the phenylalanine–tyrosine–catecholamine axis and illustrate how defects within a shared biochemical cascade can produce overlapping yet mechanistically distinct phenotypes, with direct implications for pathway-based diagnosis and treatment (Kuseyri Hübschmann et al., 2021; Davison et al., 2019; Usher et al., 2014; Zatkova et al., 2020). The following subsections examine these disorders individually, with emphasis on their molecular basis, biochemical profile, clinical presentation, and diagnostic implications.

### 3.1 PAH deficiency

PAH deficiency, classically associated with phenylketonuria, results from biallelic pathogenic variations in *PAH* that impair the conversion of phenylalanine to tyrosine. More than 1,000 pathogenic variants, including deletions, insertions, splicing defects, and missense and nonsense changes, have been associated with PAH deficiency, and most affected individuals are compound heterozygotes, with some variants occurring more frequently in specific ethnic groups (Aguirre et al., 2024b; Hillert et al., 2020; Van Spronsen et al., 2021). *PAH* encodes a tetrameric enzyme, and pathogenic variants predominantly affect the catalytic domain, although some occur at the interface of the catalytic and tetramerization domains, where they impair enzyme stability and function (Hillert et al., 2020; Flydal and Martinez, 2013). Accordingly, PAH deficiency is a loss-of-function disorder in which reduced enzymatic activity impairs the conversion of phenylalanine to tyrosine, leading to hyperphenylalaninemia and an increased phenylalanine-to-tyrosine ratio (Camp et al., 2014).

Clinically, untreated patients may present with developmental delay, intellectual disability, epilepsy, and behavioral abnormalities, whereas early-treated individuals may still show subtler neurocognitive difficulties (Van Spronsen et al., 2021; Paine, 1957). Although the mechanisms underlying neurological dysfunction are not yet fully established, elevated phenylalanine is thought to exert direct neurotoxic effects, impair myelination, and compete with other large neutral amino acids for transport across the blood-brain barrier via large neutral amino acid transporter 1 or LAT1, thereby reducing cerebral availability of tyrosine and tryptophan and secondarily affecting dopamine and serotonin synthesis (De Groot et al., 2010). Experimental studies further suggest that hyperphenylalaninemia may alter neuronal and glial development, promote oxidative stress, interfere with essential lipid metabolism, and inhibit N-methyl-D-aspartate receptor function, thereby contributing to the intellectual disability and broader neurodevelopmental phenotype observed in untreated or suboptimally treated patients (De Groot et al., 2010; Glushakov et al., 2002; Infante and Huszagh, 2001; Kienzle Hagen et al., 2002; Ushakova et al., 1997). Diagnosis is usually established through newborn screening based on dried blood spot measurement of phenylalanine by tandem mass spectrometry, followed by plasma amino acid analysis and molecular confirmation of *PAH* (Van Spronsen et al., 2021).

### 3.2 TH deficiency

TH deficiency is an autosomal recessive loss-of-function disorder caused by biallelic pathogenic variants in TH, which encodes the rate-limiting enzyme for the conversion of tyrosine to L-DOPA, the precursor of dopamine and downstream catecholamines (Willemsen et al., 2010). Most reported disease-causing TH variants are missense changes that reduce enzyme activity through impaired catalytic function, decreased protein stability or solubility, altered folding, and accelerated degradation, ultimately lowering cerebral catecholamine synthesis (Fossbakk et al., 2014). As a result, dopamine deficiency is primary, with secondary reduction of norepinephrine and epinephrine. The characteristic biochemical profile therefore includes low cerebrospinal fluid (CSF) homovanillic acid (HVA) and low 3-methoxy-4-hydroxyphenylglycol (MHPG), with normal 5-hydroxyindoleacetic acid (5-HIAA) and a reduced HVA/5-HIAA ratio; importantly, CSF HVA concentrations and the HVA/5-HIAA ratio correlate with phenotypic severity (Willemsen et al., 2010; Furukawa, 2006).

This biochemical disruption translates into a clinical continuum ranging from TH-deficient dopa-responsive dystonia, typically presenting in childhood with lower-limb dystonia and gait disturbance, to infantile parkinsonism with motor delay, and to severe infantile encephalopathy characterized by truncal hypotonia, hypokinesia, rigidity, developmental delay, oculogyric crises, autonomic features, and intellectual disability (Willemsen et al., 2010; Furukawa, 2006). These neurological phenotypes are consistent with central dopamine deficiency affecting basal ganglia motor circuits, while broader catecholaminergic depletion likely contributes to autonomic dysfunction and more complex encephalopathic presentations. Diagnosis relies on clinical suspicion, CSF neurotransmitter analysis, and molecular confirmation of biallelic TH variants (Jung-Klawitter and Kuseyri Hübschmann, 2019; Sigatullina Bondarenko et al., 2025).

### 3.3 AADC deficiency

AADC deficiency is an autosomal recessive loss-of-function disorder caused by biallelic pathogenic variants in DOPA Decarboxylase (DDC), which impair the pyridoxal phosphate-dependent conversion of L-DOPA to dopamine and 5-hydroxytryptophan to serotonin. Because dopamine is the precursor of norepinephrine and epinephrine, this defect results in a combined deficiency of dopamine, serotonin, norepinephrine, and epinephrine, generating the characteristic neurotransmitter profile of AADC deficiency (Wassenberg et al., 2017). Biochemically, the disorder is characterized by low CSF HVA, 5-HIAA, and MHPG, together with accumulation of upstream metabolites including L-DOPA, 5-hydroxytryptophan, and 3-O-methyldopa, directly reflecting the metabolic block at AADC (Wassenberg et al., 2017). This combined monoamine deficiency explains the typical phenotype, in which central dopamine depletion contributes to hypokinesia, dystonia, ptosis, and oculogyric crises, while broader serotonin and catecholamine deficiency likely contributes to developmental delay, feeding difficulties, sleep disturbances, and autonomic dysfunction (Wassenberg et al., 2017; Brun et al., 2010). Clinically, most affected individuals present in early infancy with hypotonia, movement disorders, developmental delay, and autonomic symptoms, although milder phenotypes have also been described (Wassenberg et al., 2017; Brun et al., 2010). Diagnosis is supported by cerebrospinal fluid neurotransmitter metabolite analysis, AADC enzyme activity testing where available, and molecular confirmation of *DDC*. Measurement of 3-O-methyldopa (3-OMD) in dried blood spots has also emerged as a useful diagnostic tool and a promising newborn screening approach. Genotype–phenotype correlations remain limited, and currently available biochemical markers do not reliably predict clinical severity (Brun et al., 2010; Leuzzi et al., 2014).

### 3.4 DBH deficiency

DBH deficiency is an autosomal recessive loss-of-function disorder caused by biallelic pathogenic variants in *DBH*, which block the conversion of dopamine to norepinephrine within sympathetic noradrenergic neurons and the adrenal system (Mastrangelo et al., 2023). This produces a distinctive biochemical pattern characterized by markedly reduced or absent norepinephrine and epinephrine with elevated dopamine, directly reflecting the metabolic block at DBH (Mastrangelo et al., 2023; Nagats, 1991). The resulting failure of sympathetic noradrenergic transmission explains the characteristic phenotype, in which severe orthostatic hypotension, exercise intolerance, ptosis, nasal congestion, and generalized autonomic dysfunction (Wassenberg et al., 2017). Diagnosis should be suspected in patients with profound autonomic failure and a compatible catecholamine profile and confirmed by molecular testing of *DBH* (Mastrangelo et al., 2023).

## 4 Diagnostic framework

For PAH, TH, AADC, DBH deficiencies, diagnosis is best approached through a stepwise framework integrating clinical suspicion, biochemical profiling, and molecular confirmation (Table 3).

**TABLE 3:** Integrated biochemical and molecular diagnosis techniques in phenylalanine-tyrosine catecholamine axis disorders.

Technique	Target Metabolite(s)/Genetic target	Related conditions	Sensitivity	Specificity	Limitations	References
LC-MS/MS	Phenylalanine, tyrosine, L-DOPA, dopamine, norepinephrine	PAH, TH, AADC, DBH deficiencies	Very high (pg/mL to subnanomolar range; enhanced with derivatization; low sample volume)	Very high (high molecular selectivity; minimal interference; MRM-based detection)	Requires specialized equipment and expertise; high cost; complex sample preparation; low endogenous concentrations may still pose challenges	Dikunets et al. (2020), Noh et al. (2023), Van Faassen et al. (2020), Bergmann and Schmedes (2020), Fathallah et al. (2025), Kushnir et al. (2002), Meesters et al. (2009)
HPLC (FLD/EC/fluorescence)*	Phenylalanine, catecholamines (dopamine, norepinephrine)	PAH, TH, DBH deficiencies	Moderate to high (LOD ∼0.01–0.05 μg/mL)	Moderate (susceptible to interference from structurally similar compounds and drugs)	Lower specificity than LC-MS/MS; requires careful sample preparation; limited multiplexing	Jiang et al. (2025), Peaston and Weinkove (2004), Peaston and Weinkove (2004), Li et al. (2022b), Chung et al. (2019), Dillen et al. (1986), Krstulović (1982), Punchaichira et al. (2018), Sarı et al. (2022)
Enzymatic assays	Phenylalanine	PAH deficiency	Moderate (typically μM range; suitable for screening but limited for low-level detection)	Moderate (enzyme-dependent; potential cross-reactivity)	Limited specificity; dependent on enzyme conditions; not suitable for multiplex detection	Van Spronsen et al. (2021), Smith et al. (2025), Lee (1993)
MS/MS (screening)*	Phenylalanine and related metabolites	PAH deficiency	High (μM to low μM range; high-throughput newborn screening capability)	High (accurate quantification, but limited pathway discrimination)	Limited specificity for pathway differentiation; requires confirmatory testing	Gouda and Nazim (2020), Mittal (2015), Soga and Heiger (2000)
Genetic sequencing (panels, WES, WGS)	*PAH, TH, DDC, DBH*	PAH, TH, AADC, DBH deficiencies	Very high (variant detection)	Very high (molecular specificity)	Cannot assess biochemical function; VUS interpretation challenges; may miss structural/deep intronic variants; may require LRS or functional validation	Furukawa (2006), Wortmann et al. (2022), Sankar and Vinitha (2025), Soriano-Sexto et al. (2026), Del Franco et al. (2017), Nagats et al. (2019)

* HPLC-FLD, High-Performance Liquid Chromatography with Fluorescence Detection/HPLC-EC, High-Performance Liquid Chromatography with Electrochemical Detection/MS/MS, tandem mass spectrometry.

### 4.1 Clinical suspicion and biochemical stratification

The diagnostic evaluation of disorders affecting the phenylalanine–tyrosine–catecholamine pathway should begin with clinical suspicion supported by biochemical stratification rather than sequencing alone (Hyland, 2008; Kuster et al., 2018; Rodan et al., 2015). These disorders are often suggested by combinations of movement abnormalities, developmental delay, autonomic dysfunction, hypotension, ptosis, oculogyric crises, or hypokinesia, although their presentations may overlap with other neurometabolic and neurological conditions (Wassenberg et al., 2017; Kuster et al., 2018; Rodan et al., 2015). In this setting, targeted biochemical testing helps prioritize the most likely defects and provides a pathway-based framework for interpreting subsequent molecular findings.

Initial biochemical evaluation may include CSF neurotransmitter metabolite profiling–TH/AADC/DBH deficiencies–, pterin analysis–PAH deficiency–, and selected peripheral biomarkers depending on the suspected disorder (Wassenberg et al., 2017; Hyland, 2008; Rodan et al., 2015). Current diagnostic workflows increasingly rely on LC-MS/MS and other chromatographic methods with appropriate detection systems, selected according to the analyte and clinical context (Hyland, 2008; Rodan et al., 2015). This approach is particularly useful because it can reveal disease-specific metabolic signatures, narrow the differential diagnosis, and guide gene selection for confirmatory testing (Wassenberg et al., 2017; Kuster et al., 2018). Thus, biochemical data remain central not only to the recognition of these disorders but also to the disease-specific interpretation of genomic results (Hyland, 2008; Kuster et al., 2018).

### 4.2 Molecular confirmation and genomic resolution

Once the clinical and biochemical findings are compatible with a catecholamine pathway disorder, molecular confirmation is typically pursued using targeted gene panels or broader next-generation sequencing (NGS) approaches such as whole-exome sequencing (WES) or whole-genome sequencing (WGS) (Brennenstuhl et al., 2019). These methods allow simultaneous evaluation of the core genes involved in this pathway, particularly *PAH, TH, DDC*, and *DBH*, improving diagnostic yield in patients with overlapping or atypical phenotypes (Ferreira et al., 2023). Molecular confirmation also supports treatment planning, family counseling, and recurrence-risk assessment. Sanger sequencing retains a complementary role in variant confirmation, segregation studies, and targeted testing in selected families (Arteche-López et al., 2021; Beck et al., 2016; Ng et al., 2015; Nkengasong et al., 2018; Richards et al., 2015).

In unresolved cases, additional methods may be required to detect pathogenic changes that are not adequately captured by routine short-read sequencing (SRS). These include copy-number analysis (CNV), RNA sequencing, long-read sequencing (LRS), and functional studies, which can help identify splice-altering, structural, regulatory, or other difficult-to-detect variants and support the interpretation of variants of uncertain significance (Smail and Montgomery, 2024). Accordingly, the major challenge in molecular diagnosis is not only variant detection but also accurate variant interpretation through integration of phenotype, biochemical profile, segregation data, and functional evidence (Richards et al., 2015; Smail and Montgomery, 2024).

Although NGS has substantially improved the molecular diagnosis of inherited metabolic disorders, its clinical implementation varies across platforms. In current practice, targeted gene panels and WES remain the most widely used approaches for diagnosing disorders affecting the phenylalanine–tyrosine–catecholamine axis pathway, while WGS is increasingly adopted in specialized centers. However, a proportion of patients remain without a definitive molecular diagnosis following standard SRS, often due to variants that are difficult to detect, such as deep intronic changes, structural rearrangements, or transposable element insertions (Bomba et al., 2022; Wortmann et al., 2022). In this context, emerging approaches such as LRS have demonstrated the ability to identify and characterize complex genomic variants, particularly in regions that are challenging to resolve with short-read technologies (Liu et al., 2024; Oakley et al., 2023). Nevertheless, despite their diagnostic potential, these methods are currently limited by higher costs, sequencing error profiles, and bioinformatic complexity, and are therefore primarily applied in research settings or specialized diagnostic workflows (Wortmann et al., 2022). As sequencing technologies and analytical pipelines continue to evolve, these approaches are expected to play an increasingly important role in resolving genetically unexplained cases and refining molecular diagnoses.

Despite these advances, NGS has substantially improved the molecular diagnosis of inherited metabolic disorders, but its clinical implementation varies across platforms. In current practice, targeted gene panels and WES remain the most widely used approaches for diagnosing disorders affecting the phenylalanine–tyrosine–catecholamine pathway, while WGS is increasingly adopted in specialized centers. However, a proportion of patients remain without a definitive molecular diagnosis following standard SRS, often due to variants that are difficult to detect, such as deep intronic changes, structural rearrangements, or transposable element insertions (Bomba et al., 2022; Wortmann et al., 2022). In this context, emerging approaches such as LRS have demonstrated the ability to identify and characterize complex genomic variants, particularly in regions that are challenging to resolve with short-read technologies (Liu et al., 2024; Oakley et al., 2023). Nevertheless, despite their diagnostic potential, these methods are currently limited by higher costs, sequencing error profiles, and bioinformatic complexity, and are therefore primarily applied in research settings or specialized diagnostic workflows (Wortmann et al., 2022). As sequencing technologies and analytical pipelines continue to evolve, these approaches are expected to play an increasingly important role in resolving genetically unexplained cases and refining molecular diagnoses.

### 4.3 Comparative performance of diagnostic approaches

Within this diagnostic framework, the main biochemical and molecular methods approach play complementary roles in the diagnosis of phenylalanine–tyrosine–catecholamine metabolic disorders (Table 3). Biochemical and molecular approaches play complementary roles in the diagnosis of catecholamine-related metabolic disorders. Liquid chromatography-tandem mass spectrometry (LC-MS/MS) currently represents the most sensitive and specific biochemical technique, enabling detection of catecholamines and their metabolites at picogram-per-milliliter to subnanomolar concentrations with high molecular selectivity, particularly when coupled with derivatization strategies and multiple reaction monitoring (Dikunets et al., 2020; Noh et al., 2023; Van Faassen et al., 2020). In contrast, high-performance liquid chromatography (HPLC), typically coupled with electrochemical or fluorescence detection, remains a widely used and accessible method with moderate-to-high sensitivity and good analytical performance for urinary metabolites; however, it is more susceptible to analytical interference and offers lower specificity compared to LC-MS/MS (Jiang et al., 2025; Li et al., 2022a; Peaston and Weinkove, 2004).

Genetic sequencing approaches, including targeted gene panels, WES, and WGS, provide very high specificity for establishing a definitive molecular diagnosis by identifying pathogenic variants in disease-associated genes (Wortmann et al., 2022; Sankar and Vinitha, 2025). Nevertheless, these methods do not directly assess biochemical dysfunction and may yield variants of uncertain significance, requiring careful clinical and metabolic correlation. Additionally, standard short-read sequencing may fail to detect complex genomic alterations such as deep intronic variants, structural rearrangements, or transposable element insertions, which can contribute to unresolved cases. Emerging approaches such as long-read sequencing and functional validation assays are increasingly important to overcome these limitations and improve diagnostic yield (Žigman, 2024; Soriano-Sexto et al., 2026).

### 4.4 Diagnostic access and practical limitations

Despite major advances in genomic testing, access to molecular diagnosis remains uneven across health systems. In many low- and middle-income countries, limited infrastructure, high costs, and shortages of trained personnel continue to delay diagnosis and restrict access to specialized treatment and genetic counseling (Nkengasong et al., 2018; Anticona Huaynate et al., 2015). Stepwise diagnostic strategies that combine clinical recognition, biochemical prioritization, and appropriately selected genomic methods may therefore be especially valuable in resource-constrained settings. Expanding regional sequencing capacity and collaborative diagnostic networks will be important for reducing disparities in access to diagnosis.

## 5 Therapeutics approaches

Treatment of disorders affecting the phenylalanine–tyrosine–catecholamine axis is guided by the specific enzymatic defect and may include dietary management, cofactor supplementation, neurotransmitter replacement or metabolic bypass, supportive pharmacological measures, and, in selected conditions, gene-based therapies. Clinical response varies according to residual enzyme activity, disease severity, and the timing of treatment initiation (Willemsen et al., 2010; Blau, 2013; Den Hollander et al., 2025; Qu et al., 2019; Saudubray et al., 2006).

### 5.1 PAH deficiency management

In PAH deficiency, lifelong phenylalanine restriction remains the cornerstone of treatment and is recommended for individuals with untreated phenylalanine levels >360 μmol/L, with therapeutic intensity adjusted according to age, growth, pregnancy, metabolic control, and clinical context (Smith et al., 2025; Van Wegberg et al., 2025). *PAH* genotype helps define the degree of protein dysfunction, residual enzymatic activity, and metabolic phenotype; it also has prognostic and therapeutic relevance. Patients with higher residual PAH activity are more likely to respond to sapropterin, whereas those with two null variants are not expected to benefit because residual PAH protein is absent; accordingly, contemporary classification increasingly distinguishes patients who require treatment and are cofactor responsive from those who are cofactor unresponsive (Smith et al., 2025; Van Wegberg et al., 2025; Garbade et al., 2019; Wettstein et al., 2015). In selected responsive patients, tetrahydrobiopterin (BH4) supplementation with sapropterin acts as a pharmacologic chaperone, enhances residual PAH activity, lowers blood phenylalanine concentrations, and may increase natural protein tolerance and reduce dietary burden (Qu et al., 2019).

For patients with inadequate metabolic control despite dietary treatment, pegvaliase provides an enzyme substitution strategy that bypasses the defective PAH pathway and is now an established therapeutic option in older adolescents and adults in some jurisdictions, although access and reimbursement remain variable (Smith et al., 2025; Van Wegberg et al., 2025). More broadly, current guidelines emphasize that treatment should be individualized and may involve dietary, pharmacologic, and educational modalities combined according to patient needs and preferences (Smith et al., 2025). Emerging strategies, including gene correction, gene therapy, mRNA-based therapy, and additional cofactor- or enzyme-based approaches, are under active development, with several gene therapy platforms in clinical trials; however, these approaches remain investigational, and their long-term durability, safety, and genotype-specific applicability still need to be established (Van Wegberg et al., 2025).

### 5.2 TH deficiency management

In TH deficiency, L-DOPA combined with a peripheral decarboxylase inhibitor remains the first-line treatment, but therapeutic response is highly variable and clinically relevant for both prognosis and individualized management. Patients with milder phenotypes often show a favorable response, whereas those with more severe disease may develop L-DOPA/decarboxylase inhibitor-induced dyskinesia and respond less completely, requiring careful titration and, in selected cases, consideration of alternative or adjunctive strategies such as monoamine oxidase inhibitors (Sigatullina Bondarenko et al., 2025; Wijemanne and Jankovic, 2015). Although a clear genotype–phenotype correlation has not yet been established, disease severity appears to correlate better with biochemical phenotype, as patients with more severe presentations tend to have lower CSF HVA levels, poorer response to L-DOPA, and more frequent treatment-induced dyskinesia (Sigatullina Bondarenko et al., 2025; Wijemanne and Jankovic, 2015). These findings support a personalized treatment approach based on clinical phenotype, CSF neurotransmitter profile, and tolerability rather than genotype alone. At present, no approved enzyme replacement, gene therapy, or RNA-based therapy exists for TH deficiency, and these remain areas for future research rather than established therapeutic options (Sigatullina Bondarenko et al., 2025).

### 5.3 AADC deficiency management

In disorders such as AADC deficiency, pathway-directed therapy is often complemented by supportive pharmacological management, including pyridoxine or pyridoxal phosphate, dopamine agonists, monoamine oxidase inhibitors, and multidisciplinary supportive care. However, the benefit of these measures is variable and often limited, particularly in patients with severe phenotypes, and treatment response remains difficult to predict in most cases (Wassenberg et al., 2017; Gunnell et al., 2005; Schreiber et al., 2021). Genotype–phenotype correlations in AADC deficiency are not sufficiently robust for routine prognostic use, so therapeutic decisions still rely mainly on clinical severity, treatment tolerability, and individual response rather than genotype alone (Wassenberg et al., 2017). In this context, gene therapy has become the most important mechanism-based therapeutic advance in AADC deficiency. Intraputaminal delivery of eladocagene exuparvovec, an AAV2-based vector carrying *DDC*, represents the first therapy directed at the primary underlying cause of the disorder and has shifted the field toward causal treatment (Roubertie et al., 2024). Nevertheless, its application requires a specialized multidisciplinary center, perioperative planning, and structured long-term follow-up because comparative procedural data and long-term outcome data are still limited (Roubertie et al., 2024).

### 5.4 DBH deficiency management

In DBH deficiency, droxidopa (L-threo-dihydroxyphenylserine, DOPS) bypasses the enzymatic block and restores norepinephrine production, with sustained improvement in orthostatic hypotension, exercise tolerance, and broader autonomic dysfunction when treatment is individually titrated (Robertson et al., 1991; Goldstein, 2006). Long-term follow-up data suggest that subjective response is often excellent, although renal dysfunction, anemia, hypomagnesemia, and some orthostatic features may improve only partially, supporting the need for individualized monitoring and dose adjustment (Wassenberg et al., 2021). Because DBH deficiency is extremely rare, genotype–phenotype correlations remain limited, and prognosis and therapeutic decision-making currently rely more on clinical severity, catecholamine profile, and treatment response than on genotype alone (Mastrangelo et al., 2023; Wassenberg et al., 2021). At present, no approved gene therapy, enzyme replacement therapy, or RNA-based therapeutic is available for DBH deficiency, and published treatment experience remains centered on pharmacologic norepinephrine replacement rather than disease-modifying molecular approaches (Wassenberg et al., 2021).

## 6 Translational perspectives: biosensors for future decentralized monitoring

Although biosensors are not currently part of routine clinical care for *PAH, TH, DDC,* or *DBH* deficiencies, they represent a promising translational tool for future biochemical monitoring (Table 4) (Hemdan et al., 2024; Lino et al., 2022). Their main appeal lies in enabling decentralized, real-time, and minimally invasive detection of metabolites within the phenylalanine–tyrosine–catecholamine pathway, including phenylalanine, tyrosine, L-DOPA, dopamine, and norepinephrine, thereby complementing conventional laboratory-based assays.

**TABLE 4:** Emerging biosensor platforms for relevant to phenylalanine-tyrosine-catecholamine axis disorders.

Metabolite (related disorder)	Biosensor type	Principle and method	Advantages/Limitations	Clinical feasibility/Status	References
Phenylalanine	Enzymatic/Electrochemical	Enzyme immobilization with amperometric detection via NADH* generation	High selectivity, stable, potential for point-of-care; requires enzyme preparation and stability control	Translational/POCT potential	Li et al. (2025)
	Colorimetric/Paper-based	Enzyme-coupled reaction with colorimetric readout (smartphone-compatible)	Low-cost, portable, easy to use; lower sensitivity compared to electrochemical systems	Translational/POCT potential	Robinson et al. (2016)
	Aptamer/Impedimetric	DNA aptamer binding alters electrical impedance	High specificity, rapid detection; requires electrode functionalization	Proof-of-concept (preclinical)	Idili et al. (2019)
Tyrosine (Upstream pathway relevance)	Enzymatic/Electrochemical	Nanocomposite electrodes (e.g., rGO-based) with voltammetric detection	High sensitivity and rapid response; potential nanomaterial cost and variability	Proof-of-concept (preclinical)	Kavitha et al. (2020)
	Enzymatic/Optical	Enzyme immobilization with optical detection	Biocompatible and versatile; performance may depend on biological matrix	Proof-of-concept (preclinical)	Chen et al. (2021), Li (2010)
L-DOPA (TH/AADC deficiency)	Enzymatic/Electrochemical	Carbon-based or enzyme-modified electrodes for oxidation detection	Portable, rapid detection; requires calibration and interference control	Early validation (biological samples)	Brunetti et al. (2014), Tesoro et al. (2023)
	Aptamer/Electrochemical	Aptamer-based recognition with electrochemical readout	Potential for real-time monitoring; still largely experimental	Proof-of-concept (preclinical)	Chen et al. (2024)
Dopamine (TH, AADC, DBH deficiencies)	Electrochemical (nanomaterial-based)	Nanostructured electrodes (e.g., AuNPs, graphene) with voltammetric detection	High sensitivity and selectivity; electrode fouling and interference remain challenges	Early validation (biological fluids)	Karatas et al. (2022), Li et al. (2022b), Chen et al. (2019)
	Lab-on-chip/Microfluidic	Integrated electrochemical detection in microfluidic platforms	Portable and multiplex-capable; requires multi-step preparation and validation	Proof-of-concept (preclinical)	Virdi (2023)
Norepinephrine (DBH deficiency)	Electrochemical (CNT-based)*	Carbon nanotube-modified electrodes with differential pulse voltammetry	High sensitivity (nanomolar range), applicable to biological samples; stability and reproducibility challenges	Proof-of-concept (*ex vivo*)	Rajarathinam et al. (2022)
	Enzymatic/Electrochemical	Nanoparticle-modified electrodes for voltammetric detection	Sensitive and potentially multiplexed; requires optimization and validation	Proof-of-concept (preclinical)	Huang et al. (2023)

* NADH, Nicotinamide Adenine Dinucleotide reduced form/CNT-based, Based on Carbon Nanotubes.

Recent advances in nanomaterial-based biosensors have demonstrated the feasibility of detecting catecholamines with high sensitivity and specificity (Fredj and Sawan, 2023). For example, electrochemical and voltammetric sensors have been developed for dopamine detection in biological fluids with limits of detection in the micromolar range and high analytical reproducibility, supported by nanostructured materials such as gold nanoparticles and polydopamine-modified electrodes (Karatas et al., 2022; Li et al., 2022b). Similarly, carbon nanotube-based sensors have enabled the quantification of norepinephrine in *ex vivo* tissue samples with detection limits in the nanomolar range, demonstrating potential applicability for monitoring noradrenergic dysfunction (Rajarathinam et al., 2022). These developments are particularly relevant for disorders such as tyrosine hydroxylase deficiency, aromatic L-amino acid decarboxylase deficiency, and dopamine beta-hydroxylase deficiency, in which altered dopamine and norepinephrine levels are central biochemical features.

In addition to analyte-specific sensors, integrated and portable biosensing platforms are being developed for point-of-care testing (POCT). For instance, nanomaterial-based electrochemical systems coupled with smartphone interfaces have demonstrated rapid response times, high sensitivity, and long-term stability in real biological samples, highlighting their potential for decentralized monitoring (Jędrzak et al., 2022). Furthermore, multiplexed sensor platforms capable of simultaneously measuring multiple analytes and physiological parameters in real time represent an important step toward comprehensive metabolic monitoring (Rusli et al., 2021).

Despite these advances, most biosensor technologies remain at the proof-of-concept or early validation stage, and their translation into clinically deployable systems is still limited. Several key challenges must be addressed before clinical implementation. These include ensuring analytical robustness and reproducibility across complex biological matrices, achieving sufficient specificity to discriminate structurally similar metabolites such as catecholamines and their oxidation products, and overcoming biofouling effects that reduce sensor stability and long-term accuracy (Singh et al., 2026). In addition, the establishment of standardized calibration methods, clinically validated reference ranges, and large-scale validation studies remains essential.

From a translational perspective, regulatory approval processes, device standardization, and integration into existing clinical workflows represent additional barriers. Importantly, a clear distinction must be made between experimental biosensor systems and clinically validated diagnostic platforms. In practical terms, most currently available biosensor platforms should be considered proof-of-concept or early translational systems rather than clinically deployable diagnostic tools. While current devices demonstrate promising sensitivity, rapid detection, and potential for real-time monitoring—including wearable and implantable systems—their technology readiness level remains low, and further development is required to meet clinical standards for accuracy, reliability, and reproducibility.

## 7 Methods

This article was conducted as a structured literature review designed to provide an overview of the core enzymatic defects affecting the phenylalanine–tyrosine–catecholamine axis, with a specific focus on PAH deficiency, TH deficiency, AADC deficiency, and DBH deficiency. Relevant literature was identified through searches in PubMed, Scopus, and Web of Science. Search terms included phenylalanine hydroxylase deficiency, phenylketonuria, tyrosine hydroxylase deficiency, aromatic L-amino acid decarboxylase deficiency, dopamine beta-hydroxylase deficiency, catecholamine biosynthesis, cerebrospinal fluid neurotransmitter metabolites, dried blood spot 3-O-methyldopa, whole-exome sequencing, whole-genome sequencing, long-read sequencing, and biosensors. No date restrictions were applied in order to capture both foundational studies and recent advances.

A multistep screening process was used. First, titles and abstracts were reviewed for relevance. Full texts were then assessed for inclusion if they addressed biochemical, genetic, diagnostic, therapeutic, or translational aspects directly related to the four disorders within the scope of this review or to the associated metabolic pathway. Publications focused primarily on disorders outside this final scope, articles without direct relevance to the aims of the review, and duplicate or redundant records were excluded. Reference lists of included articles were also screened to identify additional relevant publications. Approximately 250–300 records were screened, and 120–150 publications were considered directly relevant for the final narrative synthesis.

The selected literature was organized according to the structure of the review, beginning with pathway biology and proceeding to gene-specific disease mechanisms, diagnostic approaches, therapeutic strategies, and future translational perspectives. Evidence was synthesized qualitatively, with emphasis on mechanistic relevance, biochemical and molecular diagnosis, clinical applicability, and translational potential. As this work was designed as a literature review rather than a systematic review, no formal study quality assessment or risk-of-bias evaluation was performed. To improve accuracy and consistency, references were verified against their DOI or permanent URL.

## 8 Conclusion

Disorders affecting the phenylalanine–tyrosine–catecholamine axis illustrate how defects in a shared metabolic pathway can give rise to distinct but overlapping neurometabolic phenotypes. Accurate diagnosis depends on the integration of biochemical and molecular data, while effective management remains closely linked to recognition of the specific enzymatic defect. Although important advances have improved diagnosis and treatment, major challenges persist in access to specialized testing and in equitable implementation across clinical settings. Biosensor-based monitoring represents a promising future direction, but its role remains translational rather than routine. Future progress will depend on improving genotype–phenotype correlations, identifying useful prognostic biomarkers, expanding access to specialized diagnostics in low- and middle-income countries, and validating the long-term effectiveness of emerging mechanism-based therapies.
